# An update on the management of refractory cutaneous lupus erythematosus

**DOI:** 10.3389/fmed.2022.941003

**Published:** 2022-09-23

**Authors:** Alice Verdelli, Alberto Corrà, Elena Biancamaria Mariotti, Cristina Aimo, Valentina Ruffo di Calabria, Walter Volpi, Lavinia Quintarelli, Marzia Caproni

**Affiliations:** ^1^Section of Dermatology, Azienda USL Toscana Centro, Florence, Italy; ^2^Section of Dermatology, University of Florence, Florence, Italy; ^3^Rare Dermatological Diseases Unit, Department of Health Sciences, Azienda USL Toscana Centro, University of Florence, Florence, Italy

**Keywords:** cutaneous lupus erythematosus (CLE), management, anifrolumab, refractory, belimumab, rituximab, JAK inhibitors, therapy

## Abstract

Management of cutaneous lupus erythematosus (CLE) involves a combination of preventive measures, topical and systemic drugs, fairly similar for the different subtypes. Although guidelines exist, to date, no specific drugs have been specifically licensed for CLE. Antimalarials remain the first-line systemic treatment, but many patients do not respond, making refractory lupus a challenge for clinicians. The choice of alternative medication should be based on effectiveness, safety and cost. Most of the available drugs for CLE have been adapted from systemic lupus erythematosus (SLE) treatment but the existing literature is limited to small studies and evidence often lacks. As knowledge of pathogenesis of both CLE and SLE is improving, promising new therapies are emerging. In this review, we discuss the available medications, focusing on the novelties under development for CLE.

## Introduction

Cutaneous lupus erythematosus (CLE) is a chronic, autoimmune, inflammatory disease comprising several subtypes, e.g., acute CLE (ACLE), subacute CLE (SCLE), chronic CLE (CCLE) and intermittent CLE (ICLE) (1). CLE can be isolated or associated to a systemic involvement. Up to 70–80% of patients with systemic LE (SLE) develop muco-cutaneous lesions during the course of the disease and up to 25% of patients with systemic LE (SLE) show muco-cutaneous involvement at diagnosis (2, 3). Thus, a systemic involvement should always be assessed at diagnosis and at follow-up (4).

To monitor CLE progression and treatment response, two scores have been validated, e.g., the *Cutaneous Lupus Erythematosus Disease Area and Severity Index* (CLASI) and, more recently, the *Revised CLASI* (RCLASI), which are able to provide disease activity (CLASI-A) and damage (CLASI-D) in CLE patients (5, 6).

According to current guidelines (7–9), management of CLE involves a combination of topical and systemic drugs, fairly similar for the different subtypes. Although consensus over the treatment and guidelines have been succeeded over the years, to date, no specific drugs have been approved by the *Food and Drug Administration* (FDA). Most of the medications for CLE have been adapted from SLE treatment but the existing literature is limited to small studies and evidence often lacks. As drugs that have proven to be effective in systemic disease may not be effective in cutaneous disease, the treatment of refractory CLE is particularly challenging, as it is difficult to achieve a consensus on the appropriate progression of treatment beyond first- and second-line treatment options. Moreover, since many of these treatments are immunosuppressants, with possible side effects, a thoughtful approach is mandatory in order to better select the most appropriate drug (10).

General recommendations include sun protection, smoking cessation and vitamin D implementation as well as withdrawal of photosensitizing drugs and avoidance of isomorphic trigger factors (9, 11–13). Female patients are also recommended to avoid hormonal contraception containing estrogens and estrogen replacement therapies. These measures are crucial to prevent refractory CLE. In fact, studies on the photoprotective habits of lupus patients have shown an increased frequency of sunscreen utilization during years (14, 15). However, not all patients with CLE use daily sun protection, not all apply the right dose and not all re-apply sunscreen during the day. Yang et al. found that especially males, patients with dark Fitzpatrick skin types, and patients between the ages of 31–50 use less frequently sun protection than necessary (16). Accordingly, active smoking has been associated with CLE severity, with a lower risk of long-term CLE remission (17). Although it is known that it decreases the efficacy of systemic treatment, the impact of tobacco on the efficacy of antimalarials may be caused by an increase in the severity of the disease more than by resistance in smokers (18).

Topical corticosteroids remain the first-line treatment of all CLE subtypes, both in localized and widespread form (7–9). They should be applied for a short time or intermittently to reduce side effects, such as atrophy, telangiectasia and steroid-induced dermatitis. Alternatively, as first-line or second-line topical treatment, calcineurin inhibitors (0.03% or 0.1% tacrolimus and 0.1% pimecrolimus ointment) could be used, showing a better safety profile and low side effects, especially in active, edematous CLE of the face. Topical retinoids could be considered as second-line treatment in verrucous LE and other hyperkeratotic lesions of CLE, especially in cases refractory to topical corticosteroids or topical calcineurin inhibitors.

The first-line systemic treatment for all types of CLE includes antimalarials, namely hydroxychloroquine (HCQ), chloroquine (CQ) and quinacrine (Q), with HCQ being the most studied and used agent even in pregnancy and pediatric patients. However, long-term use (i.e., ≥ 5 years) and high-dose HCQ (i.e., > 5 mg/kg/day) are both risk factors for the development of HCQ retinopathy (19). Accordingly, dose should be calculated on body weight with a maximum daily dose of 5 mg/kg of real bodyweight for HCQ and 2.3 mg/kg of real bodyweight for CQ to reduce side effects. However, in contrast with current guidelines, a recent survey demonstrated that about 70% of patients uses a fixed dose of antimalarials independent of the patient’s weight. In both Europe and the USA, HCQ is often prescribed as 200 mg film-coated tablets, while 100 mg HCQ tablets are available in China, not yet approved by the US FDA. The most commonly reported daily dose of HCQ was 400 mg. An inappropriate dose of antimalarials could be one of the reasons for refractory skin manifestations (19). Antimalarials are also burdened by low therapeutic adherence (20–22), especially in younger patients and in patients not convinced of the efficacy of antimalarials in the management of their disease (19). In fact, 17.3% of CLE patients skip HCQ once a week or more often. Non-adherence to HCQ could potentially lower the risk of retinopathy in the individual patient but has been associated with an increased risk of flares and may partly explain cases of refractory CLE. Thus, in case of refractory CLE should be evaluated the adherence and eventually, dosed HCQ blood levels. The need for alternative therapies in refractory CLE has been also emphasized by the limited access to quinacrine that in recent years has restricted its combination with HCQ and CQ.

In case of refractory CLE, Q could be added either to HCQ and CQ with good results, whereas the combination of HCQ and CQ should be avoid because of the risk of irreversible retinopathy. In addition, systemic corticosteroids are recommended as first-line treatment in highly active and/or severe CLE. They should be used for short periods, gradually tapering until withdrawal, to reduce corticosteroids-associated side effects (7–9).

Second- and third-line systemic treatments include immunosuppressants and immunomodulants. Over the last years, increasing knowledges in the pathogenesis of CLE and SLE also led to several new therapeutic options, such as B-cell- or interferon (IFN) α-targeted agents. Herein we reported a review on the current drugs available for refractory CLE.

## Immunosuppressants and immunomodulants

Systemic corticosteroids are recommended as first-line treatment in highly active and/or severe CLE. Recommended second and third-line systemic immunosuppressant treatments for CLE include methotrexate (MTX), dapsone, systemic retinoids, mycophenolate mofetil and thalidomide/lenalidomide. Herein, we reported the recommended dose and summarized the evidence of efficacy.

### Systemic corticosteroids

Systemic corticosteroids are recommended as first-line treatment in highly active and/or severe CLE, in addition to antimalarials. The usual oral dose of systemic corticosteroids is 0.5–1 mg/kg bodyweight per day for about 2–4 weeks followed by tapering of the dose to a minimum (≤7.5 mg/day) with the aim to discontinue the application. During pregnancy or breastfeeding systemic corticosteroids (prednisone and methylprednisolone) should be given in a dose of not more than 10–15 mg per day (9).

Systemic corticosteroids are generally avoided in CLE patients due to the well-known side effects. However, in addition to antimalarials, they are recommended as first-line treatment in highly active and/or severe CLE (8, 9). Besides being beneficial in association to other therapies that may require time for onset of action, in a prospective, cross-sectional, multicenter study performed by EUSCLE, systemic corticosteroids showed the highest efficacy in comparison with all other systemic drugs used for CLE therapy, providing to be effective in 94.3% of the 413 treated patients. Moreover, systemic corticosteroids were most frequently (in 58.1%) and most successfully (in 96.8%) applied in cases of ACLE, probably due to the frequent association with SLE. The usual oral dose of systemic corticosteroids is 0.5–1 mg/kg bodyweight per day for about 2–4 weeks followed by tapering of the dose to a minimum (≤7.5 mg/day) with the aim to discontinue the application to avoid side-effects. In fact, LE patients are particularly susceptible to the side effects of steroids, as they are at increased risk of developing avascular necrosis at baseline (23). The continuation of treatment with antimalarials or other corticosteroids-sparing agents is recommended during the tapering and after discontinuation of systemic corticosteroids. Moreover, to reduce the risk of corticosteroids-associated side-effects, it is recommended to avoid long-term maintenance treatment with corticosteroids in CLE patients without systemic involvement. Systemic corticosteroids are also administered in association to rituximab (2 × 1,000 mg/m^2^ IV rituximab in combination with 100 mg IV methylprednisolone at an interval of 2 weeks) in patients resistant to other therapeutic agents, such as antimalarials, thalidomide, immunosuppressive drugs, and high-dose of intravenous immune-globulin (IVIg) (24).

### Methotrexate

*MTX, up to 20 mg per week, is a second-line treatment for refractory CLE, preferably subcutaneously, and in addition to antimalarials. Folic acid at a dose of 5–10 mg/week, the day after MTX injection, should be added to reduce MTX side effects.*

In a recent study on 73 patients with antimalarial-refractory CLE, MTX was found to be the second most effective alternative option after thalidomide, with fewer side effects, showing a partial or substantial resolution in 69% of the 19 treated patients (25).

In a retrospective study, 10 of the 12 analyzed patients with CLE receiving weekly administrations of 10–25 mg MTX showed significant improvements of their skin lesions within 6 weeks (26).

Another study of 43 patients with CLE, MTX, as both monotherapy and adjunctive therapy, resulted in significant improvement in activity of cutaneous lesions in 98% of patients, especially in SCLE (27). MTX was administered intravenously at initial 15–25 mg/weekly dose, then tapered to 7.5–1 5 mg/weekly in 8 patients and 10–20 mg/weekly in 7 patients. Severe side effects necessitating discontinuation of MTX treatment were recorded in seven patients (16%), solved after MTX discontinuation.

Both studies supported the use of low dose MTX for management in refractory patients.

In a retrospective study comparing MTX with MMF, MTX was successfully administrated in 72% of 18 SCLE patients and 46% of 13 DLE patient measured by CLASI improvement, with side effects reported by 28% of SCLE patients, among which nausea/diarrhea was the most common cause of discontinuation, and by 19% of DLE patients, with increased transaminases as the main cause of withdrawing (28).

Cyclosporine has been used in combination with MTX with good result and allowing lower dosing when used in combination (29).

A randomized controlled trial (RCT) study on 41 SLE patients with skin involvement comparing the efficacy and safety of MTX and CQ showed significant improvement in both groups, with no significant differences, demonstrating that low-dose MTX can be as effective as CQ (30).

Although CLE patients can benefit from MTX treatment, the drug can cause adverse sequelae, including hematologic, pulmonary, gastrointestinal, and hepatic side effects. Therefore, the drug should be administered under careful physician supervision (31).

### Dapsone

*Dapsone is the first-line treatment for bullous LE and a second-line treatment for refractory CLE, preferably in addition to antimalarials*. *Low dose treatment (50 mg daily) should be used with an increased to a maximum of 1.5 mg/kg daily based on clinical response and side effects, monitoring the glucose 6-phosphate dehydrogenase (G6PD).*

The European League Against Rheumatism (EULAR) recommended dapsone, 100 mg daily, in SLE with skin lesions, especially bullous manifestations, in non-responsive cases or cases requiring high-dose corticosteroids (32, 33).

Concerning CLE, dapsone seems to work especially on SCLE, DLE and lupus erythematosus panniculitis (LEP) (34–36).

Lindskov and Reymann treated 33 DLE patients with dapsone with satisfactory results in 48% of patients (37).

In a retrospective analysis, Klebes et al. analyzed 34 CLE patients treated with dapsone (median dose: 100 mg/day) as monotherapy or combined with antimalarials, for a mean duration of 16 months. Authors reported a complete remission in 18% (*n* = 6) of the patients and an improvement in 41% cases (*n* = 14) while in 18% (*n* = 6) patients the drug was ineffective. The best effect was seen in SCLE patients with either disease remission or improvement in 75% of the patients, similarly to other reports. Dapsone was discontinued in 4 cases due to reversible side effects and in 5 patients due to poor efficacy (38).

Coburn and Shuster treated 11 patients with DLE showing good result in 8 patients (39).

In a study by Ruzicka and Goerz on the effects of dapsone in 7 patients (4 with DLE and 3 with a wide-spread rash of SLE), SLE patients had remission of discoid lesions, oral lesions and urticarial vasculitis. However, 2 patients with SLE and generalized acute skin lesions as well as 1 patient with disseminated DLE remained unresponsive to dapsone (40). With a dapsone dose of 25 mg in combination with 500 mg vitamin C, Ruzicka and Goerz observed healing of DLE.

Successful treatment of LEP with dapsone was also seen in 11 cases. Disease remission was noted in all patients between 1 and 8 weeks (mean 4.6 weeks) (36).

Overall, the risk of dapsone-dependent side effects is very low. Dapsone is not recommended in patients with G6PD deficiency to avoid one of its severe side effects, hemolytic anemia, in these individuals. It is not recommended in individuals carrying the HLA-B*13:01 allele, which is associated to the development of dapsone hypersensitivity syndrome, a fatal side effects of this drug (41).

### Mycophenolate mofetil

*Mycophenolate mofetil (MMF) is a third-line option for refractory CLE in addition to antimalarials. Recommended starting dose is 500 mg × 2 daily, that can be increased up to 3 gr daily.*

*Mycophenolic acid (MPA) could be an alternative choice to MMF.*

MMF has been shown to be effective in different CLE subtypes, in combination with HCQ and (or) systemic corticosteroids, in small series (28, 42–44).

A prospective, non-randomized, open pilot study assessed the efficacy of mycophenolate sodium, the enteric-coated form of MMF, in 10 patients with SCLE refractory to antimalarial therapy (45). Remarkable results with significant CLASI improvement were achieved with 1.440 mg/day MMF monotherapy for 3 months. No serious side effects were reported. A retrospective analysis of 24 patients with recalcitrant CLE showed some clinical response in all patients and resolution or near resolution of disease activity in 62% of patients (46). The average final dose of MMF was 2.750 mg/day. Therapy was well tolerated and the mean time to initial response was 2.76 months. The beneficial effects of MMF in combination with HQ are highlighted in a recent case series of three patients with recalcitrant CLE. Doses of MMF from 1,000 to 1,500 mg/day were effective within 5.6 weeks (47).

### Azathioprine, cyclosporine, cyclophosphamide

Guidelines do not suggest azathioprine, cyclosporine and cyclophosphamide for CLE without systemic involvement, since few data are available in the literature with no control trials to support routine use in CLE (7–9).

Interestingly, azathioprine has proven good results in non-specific cutaneous LE manifestations, especially recalcitrant leukocytoclastic vasculitis (48).

## Retinoids

*Retinoids are a second-line treatment in selective CLE patients unresponsive to other treatment, especially hyperkeratotic lesions and verrucous LE, preferably in addition to antimalarials. The recommended daily dose of acitretin and isotretinoin in treatment for CLE is 0.2–1.0 mg/kg body weight. It usually takes 2–6 weeks for patients to achieve treatment response.*

Retinoids, including acitretin, isotretinoin and alitretinoin, have been used in refractory CLE with satisfactory results.

In a double-blinded RCT, acitretin 50 mg/day was found to be effective as HCQ 400 mg/day, with improvement or clearance of skin lesions in 48% of the patients receiving acitretin and 50% of patients receiving HCQ. However, acitretin was less tolerated (49).

Both isotretinoin and alitretinoin have been used successfully in small case series (50–52).

For verrucous LE and (or) hypertrophic lesions of CLE, sporadic case reports have also shown significant therapeutic effects of either acitretin or isotretinoin (53, 54).

The main clinical side effects associated to retinoids are skin and mucous membrane dryness, gastrointestinal symptoms, muscle weakness and arthralgia. Due to their teratogenic effects, counseling and contraception must be given to women of childbearing age. They may alter liver function and lipid profile, thus regular blood tests are mandatory.

## Immunoglobulins

Intravenous immunoglobulins (IVIG) therapy in refractory CLE has response ranging from partial to almost complete resolution of lesions (55, 56). However S2k guidelines do not suggest use of IVIG for CLE due to flare of lesions and side effects documented in various case series (57, 58).

One of the main concerns is the high cost of the treatment, which limits its widespread use.

## Fumaric acid esters (monoethylfumarate and dimethylfumarate)

Fumaric acid esters have been successfully used in CLE in small series (59, 60).

A recent open-label phase II study showed an improvement in disease activity in 11 patients receiving monoethylfumarate and dimethylfumarate, but the primary endpoint, corresponding to 50% reduction in RCLASI score, was not achieved (61). Side effects include mainly gastrointestinal symptoms, e.g., abdominal cramping, nausea, and diarrhea.

## Thalidomide/lenalidomide/iberdomide

*Guidelines recommend thalidomide as second-line treatment for refractory CLE, especially DLE and SCLE, preferably in addition to antimalarials, whereas lenalidomide is not suggested for the treatment of CLE.*

### Thalidomide

Thalidomide is an immunomodulatory, anti-inflammatory and anti-angiogenic drug, successfully used to treat CLE in severe refractory cases (62, 63). It also shows photoprotective properties, inhibiting UVB-induced keratinocytes apoptosis (64).

The first studies on thalidomide in CLE date back to 1983, when 60 patients with DLE were treated with high dose of the drug (400 mg/day), obtaining a response in 90% of cases. However, relapses after drug withdrawal were developed by nearly all patients, even if less severe (65). Subsequent case series or small sized studies reported similar results with doses of mainly 50–100 mg daily (63, 65–69).

In a Brazilian study on 65 CLE patients, 98.9% patients reported complete or partial improvement with thalidomide 100 mg daily. However, 82% of them had cutaneous relapse and 43.2% patients presented neuropathy symptoms, which limited the use of the drug (70). Similarly, a prospective study on 60 patients with refractory CLE reported a 98% clinical response rate to 100 mg of thalidomide daily, with flares in 70% patients after drug withdrawal (71). This high relapse rate was confirmed in a recent meta-analysis of 21 studies that used thalidomide for the treatment of CLE, showing a pooled response rate of 90% but a high relapse rate of 71%. After cessation of treatment, 16% of patients manifested peripheral neuropathy but only in 4% the symptoms were persistent (72).

A recent Chinese study of 69 patients demonstrated optimal response rate (71%) at 50 mg daily (73). The same dosage was administrated by Frankel et al. in 5 patients with refractory CLE, 4 (80%) of whom showed a partial or total response after 4–8 weeks of treatment (74).

Overall, thalidomide has been used primarily in the treatment of DLE and SCLE with responses in about 98% of cases: Less frequently, ACLE, LEP, LET, or non-specific lesions, such as pyoderma gangrenosum, obtained remission under thalidomide treatment, with response rate of 50%. None of the previous studies used CLASI (63). It seems that relapses generally occurred between 4 and 8 weeks after drug interruption, but all cases responded to drug reintroduction (72). The rate of relapse after thalidomide withdrawal was 71% compared with 34% with a maintenance dose. DLE forms tended to relapse most often and required a long-term maintenance dose of thalidomide while SCLE forms showed a sustained remission after withdrawal (63).

The main limitation of thalidomide in all the studies were severe side effects, especially peripheral polyneuropathy, thromboembolic events and teratogenicity (75, 76). According to meta-analyses, 24% of patients developed side effects with the need to discontinue the drug, including 16% patients with peripheral neuropathy and 2% with thromboembolic events (72).

Peripheral polyneuropathy may occur early during the first 4 weeks of treatment and is not always reversible even after the withdrawal of the drug. Low maintenance thalidomide-dose (50 mg/day) could reduce the risk of this adverse event.

In a retrospective study of 139 CLE patients, thromboembolic events were found in 8 cases. The risk was higher for patients with a history of arterial thrombosis and hypercholesterolemia. Authors recommend a starting dose of 50 mg/day of thalidomide in association with HCQ. As some patients had high anti-phospholipid antibodies (aPL) titers, low-dose aspirin could prevent thromboembolic events.

Another limitation of thalidomide is its high cost, that affects the choice of alternative drug (77).

### Lenalidomide

Lenalidomide (LND), a synthetic thalidomide analog, has proved efficient and well-tolerated in small case series of refractory CLE, both in adults and children, even after thalidomide failure, with lower side effects (78–84).

In a recent multicenter retrospective study on 40 CLE patients, mostly with concomitant systemic involvement, LND was found to be effective in 98% of the patients with a 4-point or 20% decrease in CLASI-A and a complete remission in 43% of patients (85). Authors underlined the long-term efficacy of the treatment. A median 10 month-follow-up was performed (range 07–147 months). Asthenia was the most common side effects (23% of cases) and in 12.5% of patients cardiovascular diseases and cancers were reported, leading to drug discontinuation. In another retrospective study on 19 CLE patients, of whom 12 with DLE, oral LND at starting dose of 5 mg daily, associated to an antiaggregant (acetylsalicylic acid or clopidogrel), determined a complete or partial resolution in 12 (63%) and 5 (26.5%) patients, respectively. Adverse reactions appeared in 17% cases and permanent LND withdrawal occurred in 12% of patients (86).

Totally, considering this latter study and the previous literature, 76 CLE patients (66 adults and 10 adolescents) were treated with LND with complete resolution in 88% cases, of whom 53% had a complete remission. Relapses occurred in about 26.4% (range 0–64%), especially upon dosage reduction (87).

Seven small-sized studies reported complete/partial response in all SLE/CLE treated patients with a mean time to response of 3 months. Comparing to thalidomide, LND was better tolerated with no cases of polyneuropathy or worsening of previous thalidomide-induced neuropathy. However, most of these studies did not perform nerve conduction tests. Flare rate varied from 25 to 75% occurring 0.5–10 months after drug withdrawal (87). As for thalidomide, a high teratogenicity risk was reported.

According to current studies, lenalidomide therefore appears as a valuable option in refractory CLE even after failure or limiting toxicity of thalidomide.

### Iberdomide

Iberdomide, a thalidomide derivative, may degradate Ikaros (*IKZF1*) and Aiolos (*IKZF3*), two transcription factors involved in immune cell development and homeostasis. These molecules are overexpressed in SLE and play a role in B-cell, T-cell and monocyte regulation (86). In a phase IIa study on 42 SLE, iberdomide was efficient in reducing the Physician’s Global Assessment (PGA) and CLASI-A, being a promising therapeutic strategy in CLE (88).

## Biologics and small molecules

### Targeting plasmacytoid dendritic cells and interferon signaling

Plasmacytoid dendritic cells (pDCs) are a subset of immune cells linking innate and adaptive immunity. They are well-known for being a major source of type I interferon (IFN) in response to viral infections or self-nucleic acids through signaling pathways involving pattern-recognizing receptors (PRR). pDCs have therefore a primary role in the pathogenesis of several autoimmune diseases with IFN-signature, such as lupus erythematosus. However, pDCs’ spectrum of action appears to be much wider since the description of various interactions with T, B and NK cells. In fact, the expression of proinflammatory cytokines and costimulatory molecules enhance plasma cells differentiation, antibody secretion, Tregs and Th17 lymphocytes commitment, NK cells activation and immune cells recruitment (89). Type I IFN and pDCs represent a central paradigm not only in SLE but also in CLE pathogenesis, as highlighted by lesional skin infiltration from pDCs (90). This evidence poses the basis for a potential therapeutic option in targeting IFN and pDCs in CLE.

BIIB059 is a humanized IgG1 monoclonal antibody which binds BDCA2, a pDCs’ specific receptor which inhibits the production of type I IFN. In a recent phase I, randomized, double-blind, placebo-controlled clinical trial, 8 CLE patients were treated with single doses of BIIB059 resulting in reduction in CLASI-A scores, reduced level of IFN-related genes in blood and reduced immune infiltration in skin lesions Doses were reported to range from 0.05 to 20 mg/kg. Most of the adverse events related to the drug were mild to moderate in severity, mainly consisting in upper respiratory tract infection. One treated patient developed herpes zoster on day 141 (91). A phase II trial for the treatment of SLE and CLE is currently ongoing (NCT02847598) (92).

Immunoglobulin-like transcript 7 (ILT7) is a surface molecule selectively expressed by human pDCs. VIB7734 is a monoclonal antibody properly designed to target ILT7 in order to reduce pDCs functions and count. It showed positive preliminary results in depleting circulating and lesional pDCs in CLE patients in phase I trials, with parallel improvement in disease activity and local type I IFN activity.

In two phase I studies in patients with autoimmune diseases, VIB7734 demonstrated an acceptable safety profile, comparable to that of placebo (93). Phase II clinical trial to study the treatment of moderate to severely active CLE (RECAST SLE) is now recruiting. Patients have been divided into three groups with three dosing intervals.

Among the emerging treatments for CLE, the most promising approach is represented by anifrolumab, a fully humanized, IgG1κ monoclonal antibody targeting IFN-α/β/ω receptor (IFNAR) which disrupt signaling pathways of all type I IFNs. Following the preliminary evidence of efficacy, in July 2021 the FDA approved the use of anifrolumab in SLE patients with active disease under standard therapy in USA. Contemporary, several trials investigating the efficacy and safety of the drug are ongoing in Europe and Japan (94).

A phase IIb trial comparing intravenous anifrolumab vs. placebo in SLE patients demonstrated significant improvement of cutaneous involvement in the high IFN gene signature subgroup (95). More recently, results of the second phase III RCT comparing anifrolumab 300 mg vs. placebo showed a statistically significant difference in CLASI response (49 vs. 25%, respectively, *p* = 0.039) (96). In another phase II study on the efficacy of subcutaneous anifrolumab in SLE with active skin disease, significant reductions in CLASI activity score were observed in anifrolumab groups (97).

Sifalimumab is a human igG1κ monoclonal antibody targeting IFN-α molecule. A phase IIb trial evaluated efficacy and safety of several fixed intravenous dosages in adults with moderate to severe active SLE with inadequate responses to standard-of-care treatments. Three doses’ intervals were administered to the participants (200, 600, 1,200 mg). Although the 1,200 mg dosage provided the most consistent results, no clear sifalimumab dosage effect was observed in the study. Apart from the success in reducing SLE activity, improvements in CLASI score were greater for all sifalimumab dosages compared with placebo, suggesting an interesting option for SLE and CLE. The percentages of patients with at least one adverse event, serious adverse event or adverse event leading to discontinuation were similar across the groups. The most common adverse events were worsening of SLE, urinary tract infections, headaches, upper respiratory tract infections and nasopharyngitis (98).

Besides type-I IFN, other cytokines of the interferon family are involved in CLE pathogenesis (99). Accordingly, IFNγ showed a potential central role since high levels of IFNγ mRNA were found in DLE lesional skin, while immunohistochemical analyses found statistical difference in staining of receptor between DLE skin samples and normal skin (100).

AMG 811 is a human anti-IFNγ antibody (IgG1 isotype) that selectively targets human IFNγ. The activity of AMG 811 was assessed in a phase I RCT comparing AMG 811 therapy with placebo in DLE patients, showing changes in biomarkers associated with IFNγ in the blood and skins of DLE patients. However, these findings did not reflect significant changes in CLASI score. In fact, although a single subcutaneous dose of 180 mg was well tolerated it did not lead to statistically significant improvements in any of the efficacy outcome measures (101).

CLE lesional skin showed an activation pattern of spleen tyrosine kinase (SYK), a key regulator of cell proliferation and inflammatory pathways which was suggested as a promising target for CLE treatment (102). In a double-blind Phase Ib study the maximum applied GSK2646264 dose at any time point was 10 mg/cm^2^ over 90 cm^2^ (900 mg cream containing GSK2646264 9 mg). Topical application of the SYK inhibitor GSK2646264 to active chronic and subacute CLE lesions was well tolerated over 28 days of treatment and no new safety concerns were identified. However, the trial failed to demonstrate a change in disease activity, while a modest decrease in IFN-related genes expression was found (103).

The SYK inhibitor lanraplenib (GS-9876), administered at a dosage of 200 mg, has been tested in a phase II trial in parallel with filgotinib 30 mg via oral administration in female patients with moderate-to-severe CLE, showing greater efficacy than placebo while the higher median decrease in CLASI-A was reached in the group treated with filgotinib. Most adverse events were mild or moderate in severity. Two serious adverse events were reported with lanraplenib and one with filgotinib (104).

### B cell- targeted therapies

Among B cell-targeted therapy, rituximab and belimumab have been the most studied drug in cutaneous lesions (7–9). The role of B cells in SLE pathogenesis has been well described (105), whereas their role in CLE is still controversial. A recent study by Abernathy-Close et al. identified a B cell gene signature in the skin of DLE patients, highest than in ACLE and SCLE patients and, interestingly, in patients with DLE without associated systemic disease. These data indicate that while type I IFNs are known to contribute to the recruitment and activation of B cells in autoimmune disease (106), they may not be critical drivers in the differential recruitment of B cells observed in DLE skin. Interestingly, patients with skin lesions and positive autoantibodies tend to have a lower B cell enrichment score in the skin. The role of B cell in CCLE has been also evaluated in a study conducted by Jenks et al. They reported that while most of the patients with primary CCLE are more likely to have a B cell independent disease, 38% of them exhibited a highly activated SLE-like B cell profile providing a possible marker of progression to SLE (107).

#### Rituximab

*Dosages commonly used are two 1,000 mg IV administered 2 weeks apart. Among adverse events reported to the FDA, the most common are febrile neutropenia, pyrexia, pneumonia, and anemia. Serious side effects that can lead to death, include infusion-related reactions, severe skin and mouth reactions, Hepatitis B Virus (HBV) reactivation, Progressive Multifocal Leukoencephalopathy (PML).*

Rituximab is a monoclonal antibody directed against the CD20 antigen, leading to B cell depletion.

According to current SLE guidelines, in refractory SLE or in case of intolerance/contraindications to standard immunosuppressive agents, rituximab can be introduced (30).

Concerning skin manifestations, in two large RCTs on patients with SLE (EXPLORER and LUNAR trials) rituximab failed (108, 109). However, prospective registry data showed cutaneous improvement in 70% of rituximab-treated patients with a partial or complete remission of mucocutaneous lesions (107). Study findings suggest that rituximab may be effective in treating severe CLE in some patients with systemic disease, especially those with acute and non-specific types (110). Bullous lupus and LEP have also improved after rituximab (111–116).

Recently, Mumford et al. reported the resolution of refractory isolated DLE with rituximab, suggesting a possible role of B-cell even in this subtype of CLE (117).

Thus, rituximab may have efficacy in patients with SLE and severe active CLE; however, outcomes may vary with SCLE and CCLE subtypes and may reflect the variation in co-medications (93). Its use could be considered when treating severe CLE in some patients with systemic disease, especially those with acute and non-specific types.

#### Belimumab

*The recommended dose for SLE and lupus nephritis is 200 mg once weekly, administered subcutaneously, regardless of weight. Therapy should be interrupted after 6 months if no improvement is obtained. Adverse reactions more frequently reported* (> *5% of SLE patients) were viral infections of superior respiratory tract, bronchitis and diarrhea.*

Belimumab is a monoclonal antibody that reduces B lymphocyte survival by blocking the binding of soluble human B lymphocyte stimulator (BLyS) to its B cell receptors.

It is approved for SLE whereas no clinical trials have formally studied its effect on CLE (32).

The S2K guidelines do not recommend the use of belimumab for CLE (9); on the contrary, Lu et al. suggested belimumab as fourth-line treatment for widespread, refractory CLE lesions in patients with active SLE, especially those who have repeated recurrence of ACLE lesions during tapering of systemic corticosteroids (8). Accordingly, in a *post hoc*, pooled analysis of two phase III trials on belimumab in SLE (BLISS-52 and BLISS-76) the treatment, in combination with standard therapy, was associated with statistically significant improvement in mucocutaneous manifestations vs. placebo as assessed by both Safety of Estrogens in Lupus Erythematosus National Assessment– Systemic Lupus Erythematosus (SLE) Disease Activity Index (SELENA-SLEDAI) and British Isles Lupus Assessment Group (BILAG) scale (118). CLASI was not validated until 2011 and therefore was not studied in these randomized controlled trials.

Belimumab was associated with significant improvements in maculopapular eruption (mild), alopecia and active discoid lesions (119).

Recently, a study on 67 Italian SLE patients treated with belimumab, including 19 with mucocutaneous involvement, demonstrated a significant reduction of median CLASI activity score at 24 months, from 5 (range 1–14) to 0.5 (0–6) (120).

Vashisht et al. reported a dramatically improvement of median CLASI activity scores [from 17 (range: 9–31) to 3 (range 2–14); (*p* = 0.043)] in 5 patients with SLE with recalcitrant CLE after belimumab (121).

Dresco et al. also found a significant clinical improvement in 83% out of 7 patients with CLE with or without SLE, based on the CLASI and RCLASI activity score as well as their quality of life (DLQI) (122).

In a multicentric, retrospective observational study on 16 patients with CLE, of whom 13 with concomitant SLE, 50% of cases responded to belimumab, administered intravenously at 10 mg/kg every 2 weeks for 3 doses and then monthly, with a reduction in CLASI score, although an overall statistical improvement was not observed. Authors suggested that belimumab may be beneficial in some patients, mostly those with mild persistent activity and phototypes IV to VI. Interestingly, a clinical response was observed in all the 3 patients with isolated CLE (123). However, to date, the evidence about the effectiveness of belimumab in CLE not associated with SLE is scarce. Only isolated refractory cases of CLE successfully treated with belimumab have been recently reported.

### Janus kinase inhibitors

*Ruxolitinib or baricitinib (JAK1/JAK2 inhibitors) and tofacitinib (primarily JAK3 inhibitor), have been reported to clear recalcitrant CLE lesions. Commonly reported adverse effects are infections associated with herpes virus (herpes simplex labialis, reactivation or primary infection with varicella zoster virus, VZV), nasopharyngitis, as well as infections of upper respiratory tract and urinary tract. Manifestation of acne and gastrointestinal side effects, such as nausea and diarrhea, have also been observed. For topical applications, acne and pruritus have been described. Furthermore, patients treated with JAK inhibitors should perform strict contraception until at least 1 week after the end of treatment* (124).

The Janus kinases (JAKs) are intracellular tyrosine kinases involved in a broad variety of inflammatory cascades participating in the pathogenesis of both SLE and CLE (107). Particularly, interferon-associated JAK activation is thought to play a key role in CLE lesions, since a significant upregulation of JAK signaling in cutaneous lesions was demonstrated (125).

Two studies have described the use of JAK inhibitors in the treatment of CLE, using the SLE Disease Activity Index 2000 as the main end point which is not specific to skin disease (126, 127). Baricitinib showed complete remission of a refractory papulosquamous rash in an SLE patient (128) and complete clearance of subacute CLE and no further progression of the FFA in a patient who was started on baricitinib 4 mg for 2 months, followed by ongoing maintenance therapy with baricitinib 2 mg (129).

Ruxolitinib, at the full dose of 20 mg twice daily, baricitinib and tofacitinib have been trialed as therapeutic options for familial chilblain LE (130–135). Elman et al. also reported successful response to tocilizumab in non-familial refractory chilblain LE (136).

Bonnardeaux and Dutz showed an improvement in CLASI score in 3 patients with different refractory CLE subtypes treated with tofacitinib administered orally at a dosage of 5 mg twice daily (137). Moreover, topical tofacitinib 2% ointment was found to solve recalcitrant periorbital DLE in a case report (138).

## Targeting cytokines and their receptors

### Ustekinumab

Ustekinumab is a monoclonal antibody targeting IL-12 and IL-23. Although it seems to be effective in SLE, its role in the management of CLE is still debated (139). Few case reports of successful treatment of SCLE and DLE with ustekinumab, administered at a dosage of 45 mg or 90 mg with subcutaneous injection as for psoriasis (140–142) were reported in literature, while in a recent phase II RCT, ustekinumab in addition to standard therapy resulted superior to placebo in SLE patients with a baseline CLASI-A ≥ 4, showing a 50% improvement of CLASI-A in 17/32 (53%) patients under ustekinumab vs. 6/17 (35%) of the placebo group (*p* = 0.032) (143). However, the extension study involving 24 subjects in ustekinumab group vs. 14 patients under placebo showed > 50% improvement in CLASI-A score in 79 and 100% of the subjects groups, respectively, at week 112 (144). Moreover, some reports of ustekinumab-induced SCLE are available, generating debate over its use in CLE (145, 146).

### Low-dose IL-2

In a recent phase II study of 40 SLE Chinese patients receiving a 12-week treatment with 1 million IU subcutaneous IL-2, skin lesions and alopecia improved according to SELENA-SLEDAI and BILAG scores. However, assessment of disease activity with CLASI score was not performed (147, 148).

## Conclusion

Current treatment regimens for CLE generally comprise antimalarials, systemic corticosteroids, immunosuppressive and immunomodulant drugs, while cytotoxic agents are reserved for severe cases. However, available drugs are not always effective and side effects may occur following long-term use. Moreover, chronic steroid exposure and wide spectrum immunosuppression are major triggers of organ damage. As the skin greatly contributes to the burden of disease in terms of personal and psychological wellbeing, occupational disability and therefore medical and social costs, the development of new treatment protocols for severe and refractory cases is necessary.

In the last years, research on the pathogenesis of SLE and CLE had improved, and several new biologics and small molecules-based treatments have been proposed with promising results on skin disease. However, the lack of large clinical data and of standardized and homogeneous score to assess disease activity such as CLASI and RCLASI is a major impediment to improve management strategies in CLE.

Therefore, future prospective studies on this field should be proposed, with the contribution of expert dermatologists.

## Author contributions

AV concepted the whole work, drafted and submitted the manuscript. AC, EM, CA, VR, WV, and LQ contributed to literature revision, manuscript production, and pictures collection. MC concepted the work and revised carefully the manuscript. All authors contributed to the article and approved the submitted version.
